# The Intestinal Fatty Acid-Enteroendocrine Interplay, Emerging Roles for Olfactory Signaling and Serotonin Conjugates

**DOI:** 10.3390/molecules26051416

**Published:** 2021-03-05

**Authors:** Jocelijn Meijerink

**Affiliations:** Division of Human Nutrition and Health, Chair Nutritional Biology, Wageningen University, Stippeneng 4, 6708 Wageningen, The Netherlands; jocelijn.meijerink@wur.nl

**Keywords:** olfactory receptor, 5-HT, GLP-1, enteroendocrine, enterochromaffin, gut, *N*-acyl serotonines, short chain fatty acids, microbiota

## Abstract

Intestinal enteroendocrine cells (EECs) respond to fatty acids from dietary and microbial origin by releasing neurotransmitters and hormones with various paracrine and endocrine functions. Much has become known about the underlying signaling mechanisms, including the involvement of G-protein coupled receptors (GPCRs), like free fatty acids receptors (FFARs). This review focusses on two more recently emerging research lines: the roles of odorant receptors (ORs), and those of fatty acid conjugates in gut. Odorant receptors belong to a large family of GPCRs with functional roles that only lately have shown to reach beyond the nasal-oral cavity. In the intestinal tract, ORs are expressed on serotonin (5-HT) and glucagon-like-peptide-1 (GLP-1) producing enterochromaffin and enteroendocrine L cells, respectively. There, they appear to function as chemosensors of microbiologically produced short-, and branched-chain fatty acids. Another mechanism of fatty acid signaling in the intestine occurs via their conjugates. Among them, conjugates of unsaturated long chain fatty acids and acetate with 5-HT, *N*-acyl serotonins have recently emerged as mediators with immune-modulatory effects. In this review, novel findings in mechanisms and molecular players involved in intestinal fatty acid biology are highlighted and their potential relevance for EEC-mediated signaling to the pancreas, immune system, and brain is discussed.

## 1. Introduction

Fatty acids from our diet and those produced by the intestinal microbiota are not only pivotal as an energy source and molecular building blocks, but also serve as signaling molecules in many biological processes, either directly or via their metabolites. Throughout the gastrointestinal (GI) epithelium, fatty acids and their derivatives are recognized by specialized enteroendocrine cells (EECs) mainly via G-protein coupled receptors (GPCRs). Activation of these receptors triggers the release of peptide hormones and neurotransmitters that exert many key functions to control gut metabolism and beyond [1]. In recent years, much has been learned about the diverse signaling cascades and mechanisms that convey information resulting from chemosensing of fatty acids and other nutrients to the brain, pancreas, immune system, and other tissues.

This review describes evolving research pointing at the significance of odorant receptors (ORs) as fatty acid sensors and at the potential role of fatty acid conjugates, both as part of the intestinal enteroendocrine signaling cascade. The GPCR subfamily of odorant receptors was originally considered of interest only for oral-nasal perception of volatile compounds. Meanwhile, it has become clear that functional ORs are expressed in many non-olfactory tissues including testis, prostate, kidney, adipose tissue, muscle, skin, hair, immune cells, heart, pancreas [2,3,4,5,6,7,8], and the GI tract [9,10,11,12,13,14,15]. Here, they play a role in diverse physiological processes varying from sperm chemotaxis, modulation of cancer cell proliferation, blood pressure regulation and immune cell migration to adiposity, gut hormone release, and modulation of energy- and insulin metabolism [3,5,9,14,16,17,18,19,20,21,22]. Throughout the body they act as chemosensors for endogenous molecules that have only partly been identified thus far. Emerging evidence suggests that within the intestine, ORs can recognize nutrients and microbial metabolites, such as short chain fatty acids (SCFAs), medium chain fatty acids (MCFAs), as well as branched chain fatty acids (BCFAs), and modulate glucagon-like-peptide-1 (GLP-1) and serotonin (5-HT; 5-hydroxytryptamine) signaling by EECs. Interestingly, the OR subfamily of GPCRs is amongst the largest in our genome [23] and most ORs are still classified as orphan receptors [24]. This holds particularly true for the GI tract, where only a limited number has been characterized.

Next to fatty acids themselves and their oxygenated derivatives, several of their esters and conjugates with amines have been discovered as an important group of signaling molecules throughout the body [25,26,27,28,29,30]. These include the well-known group of the *N*-acyl-ethanolamides [31,32,33,34,35,36] and 2-acyl glycerols [37], of which several are classified as endocannabinoids. More recently, fatty acid conjugates with serotonin (5-HT) emerged as a class of molecules with immune-modulatory and anti-oxidant effects [38,39]. Of these, the 5-HT acyl-conjugates with long chain fatty acids (LCFAs) are present in the GI tract and the available evidence points towards a role in modulating immune responses and relevance for inflammatory pathologies of gut [40,41]. In relation to these lines of research, specific attention is paid to the central role of serotonin. Despite its versality, it is remarkable that so many knowledge gaps remain regarding the physiology of serotonin in the gut.

## 2. Intestinal Enteroendocrine Cells and Their Main Products

The gastrointestinal tract consists of an epithelial lineage of different cell types that can be distinguished based on their functionality. The EECs comprise only 1% of the intestinal lineage, but together they form one of the largest endocrine tissues of the body [42,43]. Enteroendocrine cells produce over 20 different gut hormones bearing diverse roles, which are still far from fully understood [44,45]. Among these, probably the best studied are GLP-1 and glucose-dependent insulin tropic polypeptide (GIP), both incretin hormones involved in the induction of glucose-dependent insulin secretion from pancreatic cells in response to nutrients [1,46,47,48]. Two other key hormones that play important roles in GI physiology are cholecystokinin (CCK) and plasma peptide YY (PYY; peptide tyrosine tyrosine). CCK exerts diverse physiological functions; it stimulates the release of bile acids and pancreatic enzymes, enhances gut motility, reduces food intake, and is involved in lipid transport [49,50,51]. PYY plays a role in appetite regulation but emerging evidence also points to a prominent role in pancreatic functioning [52]. EEC subtypes are distinguished and traditionally classified based on their location and main secretory hormones [44]. The composition of hormones released varies along the proximal to distal GI tract, being high for CCK and GIP in the proximal parts, whereas GLP-1 and PYY are predominantly produced in the distal ileal and colonic GI tract [44,53]. Accumulating evidence based on both transcriptome profiling as well as protein studies in mice shows overlapping patterns of hormone secretion and expression by the different subtypes, suggesting that the current classification is not fully accurate and likely far more complicated [54,55,56,57,58,59,60,61].

A somewhat distinct enteroendocrine cell is the serotonin producing enterochromaffin cell (EC), which in the small intestine co-expresses, although to a lesser extent, GLP-1 and CCK, in addition to 5-HT [58]. Its expression profile is dominated by Tph1, coding for tryptophan hydroxylase 1 (TPH1), the enzyme responsible for 5-HT production in ECs [62]. Enterochromaffin cells make up a large part of the total EEC population in the GI tract (SI) and are the most abundant EEC sub cell type in the colon [61,63]. Serotonin, of which more than 95% of the total body production originates from the intestinal tract, is involved in diverse physiological functions like intestinal motility, platelet function, energy metabolism, and immune modulatory processes [64]. Additionally, ECs have been suggested to act as sensors for irritants conveying information on pain via the gut brain axis by serotonergic (calcium) signaling [12].

EECs release peptide hormones by activation of nutrient sensing GPCRs expressed on their surface. EECs express GPCR taste receptors for sweet (TAS1R2-TAS1R3), umami (TAS1R1-TAS1R3), bitter (TAS2R family), and fat (FFAR1 (GPR40), FFAR2 (GPR43), FFAR3 (GPR41), FFAR4 (GPR120)) and GPR119 [65,66,67,68]. Besides, more recently the functional relevance of EEC-expressed chemosensors belonging to the G-protein coupled olfactory receptor gene family (with individual receptors named OR for human and Olfr for mice) has been evidenced [8,69].

## 3. Diet-Derived Bioactive Fatty Acids in the Gut

### 3.1. Digestion and Microbial Production of Diet-Derived Bioactive Lipids in the Gut

Dietary fat consists predominantly of triacylglycerols (TAGs) and accounts for 90% to 95% of the total energy derived from fat in the diet. Digestion of TAGs takes place in the upper part of the jejunum by activity of pancreatic lipase, resulting in the release of 2-monoacylglycerols (2-MAG) and free fatty acids (FFAs) [70]. Long chain fatty acids (LCFAs), which carry a C-atom tail of 12 or more, are taken up by protein facilitated transfer by the membrane-bound glycoprotein CD36 and fatty acid binding protein 5 (FABP5), or by passive diffusion [70]. Once inside the enterocytes, they are re-esterified and packaged into chylomicrons and further transported via the lymphatic system to the blood circulation [70,71]. The mono-unsaturated LCFA, oleic acid, a main constituent of olive oil, is also converted by enterocytes into the lipid messenger oleoyl ethanolamine (OEA) (see Section 3.3). The main dietary polyunsaturated LCFAs, linoleic acid (18:2n-6), α-linolenic acid (ALA, 18:3n-3), eicosapentaenoic acid (EPA; 20:5n-3), and docosahexaenoic acid (DHA; 22:6n-3) are essential fatty acids and should be obtained from the diet. They cannot be formed in mammals as they lack enzymes to insert a double bond in the *n* − 6 or *n* − 3 position [32,72,73]. ALA is present in substantial quantities in vegetables, nuts, flaxseed (linseed), and some vegetable oils, while DHA and EPA can be mostly found in fatty fish [32,71,74]. Although EPA and DHA can be formed out of ALA, their formation is, particularly in adults, very limited [75]. Medium chain fatty acids (MCFAs), which by definition consist of 6 C to 12 C atoms, are present at low concentrations in butter, milk, yogurt, and cheese, but can be found more abundantly in coconut oil and palm kernel oil [71]. Medium chain fatty acids and short chain fatty acids (SCFAs) seem to directly pass the mucosal membrane and enter the blood stream via intestinal capillaries [68,76].

The intestinal microbiota mainly resides within the distal part of the GI tract where it interacts with the intestinal epithelial layer. Short chain fatty acids, which are saturated aliphatic acids with fewer than six carbon atom tails [68], are primarily formed by the microbiota of the colon after fermentation of undigestible carbohydrates and dietary fibers, although ileal flora also accounts for a small percentage of SCFA production in the gut. Particularly butyrate, but also propionate and acetate exert diverse pivotal physiological roles. Butyrate serves as primary fuel for intestinal colonocytes, and has anti-inflammatory and anti-carcinogenic properties [77]. It is present at micromolar concentrations in the intestinal lumen and utilized locally, in contrast to propionate and acetate, which drain into the portal vein [77]. Propionate is metabolized by the liver and thought to be involved in keeping blood pressure balance [5], while acetate circulates in blood and is able to cross the blood brain barrier [77]. Branched chain fatty acids (BCFAs), such as isobutyrate, 2-methylbutyrate, and isovalerate can be formed as a result of protein fermentation, in particular in situations where there is a shortage of dietary fibers, e.g., with low vegetable intake.

### 3.2. Fatty Acid Sensing Receptors Expressed on EECs and ECs

Free fatty acids (FFA) interact with FFA-sensing GPCRs expressed on EECs scattered throughout the epithelial lining [66]. The different free fatty acid receptors (FFARs), FFAR1-4, show ligand specificity dictated by the aliphatic chain length of the FFA-carbon tail, albeit some partial overlap exists particularly for fatty acids with long carbon atom tails [68,71]. FFAR1 and FFAR4 respond mainly to LCFAs, while FFAR2 and FFAR3 sense SCFAs. To be more detailed, FFAR1 (GPR40) responds to MCFA and particularly to LCFAs, thereby displaying IC50 values in the low micromolar range. In humans, FFAR1 exerts the highest sensitivity for omega-3- and omega-6 unsaturated LCFAs (particularly DHA and EPA), oleic acid, and the saturated fats lauric acid, myristic acid, and palmitic acid [68]. FFAR4 (GPPR120) seems more specific for unsaturated LCFAs, displaying the lowest IC50 values for linoleic acid (18:2n-6), α-linolenic acid (ALA, 18:3n-3), γ-linolenic acid (C18-3n:6), and palmitoleic acid (C16:1n-7) [68].

The SCFA butyrate is a ligand for the niacin/butyrate receptor GPR109A, while acetate and propionate particularly activate FFAR2 (GPR43). Butyrate also activates FFAR2, but in humans its potency is less compared to that of acetate and propionate [68]. Out of the SCFAs, valerate is the most potent ligand for FFAR3 (GPR41).

Recently, members of the olfactory GPCR subfamily, namely Olfr78/OR51E2 and Olfr558/OR51E1 have been identified as sensors for SCFAs and/or BCFAs in the gut. Olfr78 (mouse) and OR51E2 (human) were found to be responsive to propionate and acetate [5,78] and Olfr78 was shown to detect physiological concentrations of lactate [79]. Although Olfr78 seems far less sensitive to propionate and acetate compared to FFAR2, it has been indicated to be of physiological significance [5]; see Section 5.1). Interestingly, identification of biologically relevant ligands for OR51E2, which was also found to be a key marker for prostate cancer, revealed that out of the 2500 virtually screened and subsequently 55 experimentally validated compounds [16], several endogenous compounds could activate OR51E2. Out of these, palmitic acid and *N*-acetyl glutamic acid seem of particular interest for EEC signaling.

Olfr558 (mouse) responds to the branched chain fatty acid bacterial metabolites isovaleric acid, and to a much lesser extent to isobutyrate and butyrate [12]. Out of a screening of 5000 compounds, the human orthologue of Olfr558, namely OR51E1 (also called Dresden(D)-GPCR), displayed the highest sensitivity for 3- and 4- methyl-valeric acids and lower sensitivity to valeric acid, isovaleric acid, and 2-methyl-valeric acid [80]. Additionally, nonanoic acid has also been found to be a ligand for OR51E1, exhibiting similar potency as isovaleric acid [81]. Interestingly, Adipietro et al. [82] compared a number of human ORs with their orthologues in primates and mouse and revealed that although the ligand profile can be similar, the potency and efficacy frequently differs dramatically between the different orthologues [82]. Table 1 summarizes the known ligand profiles of GPCRs expressed on EECs and ECs that interact with free fatty acids.

The canonical signal transduction cascade in the olfactory system comprises the Gα_olf_ protein that is activated upon specific ligand stimulation of ORs. Further downstream signaling involves the subsequent stimulation of adenylyl cyclase III (ACIII; also known as ADCY3), which is followed by a rise in cytoplasmic cAMP levels and a successive influx of sodium (Na^+^) and calcium (Ca^2+^) by opening of nonspecific cation- selective cyclic nucleotidegated (CNG) channels [8]. Studies in ECs in intestinal organoids and pancreatic α-cells report the involvement of the Gα_olf_/s-adenylyl cyclase signaling cascade for fatty acid-induced 5-HT secretion and azelaic acid (nonanedioic acid)-mediated glucagon secretion, respectively [12,22]. However, alternative signaling cascades have been indicated for olfactory chemosensing in non-oral nasal tissues as well [8,20,21].

### 3.3. Oleoyl Ethanolamine, a Bioactive Lipid Conjugate with a Key Physiological Role in the Gut

Fatty acid conjugates are known to act as signaling molecules in a plethora of physiological processes. Amongst them, the class of fatty acid conjugates with ethanol amine, the fatty acid ethanolamides (FAEs), is now widely recognized for its diverse biological functions. In the GI tract, the oleic acid-derived constituent, oleoyl ethanolamine (OEA), is particularly documented for its pronounced effects, including those acting via the gut-brain axis [33]. OEA has been shown to enhance satiety by prolonging meal intervals. This effect is dependent on luminal levels of its fatty acid precursor oleic acid in the proximal GI tract [31]. Cellular uptake of oleic acid by small-intestinal enterocytes is facilitated by the membrane bound glycoprotein CD36, whereafter oleic acid is firstly converted to *N*-oleoyl-phosphatidylethanolamine (NOPE), a member of the *N*-acylphosphatidylethanolamine (NAPE) family of membrane phospholipids, and finally to OEA [31,83]. Numerous investigations in both animals and man have gained substantial evidence that OEA exerts its anorectic effects via the nuclear receptor peroxisome proliferator-activated receptor alpha (PPARα) [31,84], through which it additionally enhances energy expenditure and reduces fat mass [85]. OEA signaling via the gut-brain axis mainly takes place via afferent vagal fibres. It seems that in the central nervous system (CNS), histamine and neuropeptide oxytocin are involved in further conveying of OEA-induced satiating effects [84]. Interestingly, OEA modulates also dopaminergic neuronal reward circuits; CNS dopamine release was found to be disturbed in obese animals and could be restored by adding OEA [86]. OEA is also a low micromolar ligand for the G-protein coupled receptor, GPR119, which seems to be expressed both on the luminal and basolateral side of EECs [1,86]. GPR119 is also activated by the ester of oleic acid with glycerol, 2-oleoyl-*sn*-glycerol (2-OG) by 2-MAG and many other lipid conjugates (see Table 1) [87]. Although still far from understood, it is now thought that GPR119 acts as a luminal sensor of ingested fat only, as in GPR119 knockout (KO) animals, OEA-induced satiety was not altered [88]. GPR119 is also expressed in pancreas and in hepatocytes and seems to reduce obesity-induced NASH in the liver.

## 4. The Fatty Acid-GLP-1-Pancreas Triangle

### 4.1. Fatty Acid Signaling by Enteroendocrine L Cells

The distally located classical enteroendocrine L-subtype predominantly expresses GLP-1 aside to PYY and oxyntomodulin (OXM) [1]. GLP-1 and PYY are secreted by enteroendocrine L-cells upon stimulation of dietary nutrients or microbial metabolites. Intestinal produced GLP-1 exerts its function both in an endocrine and paracrine way. GLP-1 that reaches the blood circulation induces insulin release from the pancreas but only in the presence of orally induced glucose, which is generally referred to as incretin effect [46]. In addition, GLP-1 is known to regulate gastric emptying and influence food intake, effects likely mediated via afferent nerve fibers.

Knockout studies in mice that specifically abrogated distally produced GLP-1 demonstrated that circulating GLP-1 originates for 90% from the distal GI tract and functions in the regulation of pancreatic insulin release and gastric emptying [47]. Although animal data cannot be fully translated to humans and the precise role of GLP-1 in humans is still not fully elucidated, the relevance of intestinal produced GLP-1 for insulin regulation in humans is underscored by the success of GLP-1 based drugs in clinical settings with diabetic patients [1].

Numerous studies have contributed to the view that SCFAs of gut microbial origin can induce GLP-1 release from enteroendocrine L cells. Particularly, KO studies in mice have provided evidence that FFAR2 and to a lesser extent FFAR3 is involved in this signaling [89]. Next to FFAR2 and FFAR3, FFAR1 and FFAR4 have also been shown to be involved in GLP-1 signaling [90,91]. FFAR1 and FFAR4 sense MCFAs and poly unsaturated long chain fatty acids. Besides, many other GPCRs, like GPR119, have been shown to induce GLP-1 release from EECs. It is thought that in the proximal GI tract, some GPCRs (FFAR1, GPR119) are located at the basolateral side of the membrane of EECs, and not luminally as traditionally assumed, where they are stimulated by FFAs that have been first absorbed by enterocytes and subsequently released by chylomicrons [92,93]. In this way they might inform the body about the presence of high energy nutrients. As FFA signaling via GPCRs including FFARs is the topic of several excellent recent reviews, including but not limited to [1,68], in this context, particularly new developments in the field of OR signaling will be further highlighted.

### 4.2. Microbial-Metabolite OR Sensing in GLP-1/PYY Signaling

In light of the EEC/GLP-1 pancreatic signaling axis, emerging evidence points to the potential relevance of chemosensing via receptors belonging to the GPCR subfamily of odorant receptors. Amongst OR genes in colon, OR51E2 was found to be the highest expressed OR gene, as determined by high-throughput mRNA sequencing (RNA-Seq) [4]. Although OR51E2 is expressed in many other tissues as well, the colon was found to exhibit the second highest expression out of 16 other non-gut derived human tissues examined [4]. OR51E2 has been reported to sense the microbial-derived metabolites acetate and propionate. In colon, cells staining positive for Olfr78 (the mouse orthologue of OR51E2), showed co-localization with PYY-stained cells and to a lower extent with GLP-1 stained cells [94]. However, no co-localization was found with 5-HT stained cells. The latter seems contradictory to the findings by Lund et al. [95], who reported that Olfr78 is the highest enriched receptor in 5-HT-excreting EC cells (see Section 5.1).

In young pigs, OR51E1, the human and porcine orthologue of mouse Olfr558, has been shown to be expressed along the full length of the GI tract. Remarkably, an approximately 4 to 5 times higher expression of OR51E1 was found in the duodenum and in the stomach, respectively, when compared to other parts of the small and large intestine [96]. Using immunostainings, it was shown that OR51E1 was almost completely co-localized with chromogranin A (ChgA), suggesting it relevance for entero-endocrine signaling. Interestingly, a high percentage of PYY- and 5-HT stained cells did co-localize with OR51E1 [96]. Co-localization of OR51E1 was also found with GLP-1 and olfactory marker protein (OMP) in the enteroendocrine L-cell line NCI-H716, which is originally derived from cecum. GLP-1 and PYY induced excretion by nonanoic acid was shown to be mediated via OR51E1, as siRNA of OR51E1 attenuated secretory effects [97]. In human ileum, GLP-1 expression co-localized with that of OMP [97]. OR51E1 responds to several ligands, including butyric acid and nonanoic acid [81], but seems most sensitive to the branched chain fatty acids, 3- and 4- methyl-valeric acids [80]. All together, these studies point to a potential role for OR51E2 and OR51E1 in intestinal EEC signaling. Table 1 summarizes GPCRs responsive to free fatty acid ligands with a potential role in GLP-1/PYY signaling in intestinal EECs.

### 4.3. Fatty Acid OR Signaling in the Pancreas

A few reports have pointed to a potential role of fatty acid mediated OR signaling in pancreas (see Table 2). The pancreas, being essential for glucose metabolism, harbors β cells and α cells that secrete insulin and glucagon, respectively, to regulate and maintain blood glucose homeostasis. Octanoic acid (also called caprylic acid), a medium-chain fatty acid, could potentiate glucose-stimulated insulin secretion via Olfr15 in pancreatic β cells [20,21]. Interestingly, these effects were impaired in islets from diabetic leptin receptor deficient mice (db/db) and mice kept on a high fat diet (HFD). In addition, expression levels of Olfr15 were largely reduced in both db/db and HFD mice [20]. Olfr15 expression was found to be specific for pancreatic islets and to uniformly co-express Olfr821 [21]. Notably, octanoic acid-induced effects via Olfr15 were not mediated via the Gα_olf_-cAMP-PKA pathway, but rather by increasing intracellular Ca^2+^ through the phospholipase C (PLC)-inositol triphosphate (IP3)-dependent pathway [20,21].

Furthermore, several Olfrs, namely Olfr544, Olfr543, Olfr545, and Olfr1349 have been identified in glucagon secreting pancreatic αTC1-9 cells. Next to this, olfactory marker protein (OMP), adenylyl cyclase III (ACIII), and olfactory G-protein (Gα_olf_), components of the canonical olfactory signaling cascade were found to be expressed in pancreatic mouse tissue [22]. OMP co-localized with the glucagon-producing α-cells, but not with insulin or somatostatin positive cells, which seems in accordance with the non-canonical signaling cascade as uncovered for Olfr15 in pancreatic β cells. Azelaic acid, a known ligand for Olfr544, induced glucagon release in αTC1-9 cells, which could be blocked by Olfr544-specific siRNAs [22].

Out of the FFARs (1–4), a role of particularly FFAR1 has been demonstrated in pancreatic signaling. In addition, GPR119 is involved in pancreatic signaling of insulin secretion by pancreatic β cells [98].

## 5. Serotonin Signaling by Enterochromaffin Cells

### 5.1. The Fatty Acid-5-HT Interplay

As introduced in Section 2, ECs in the gut account for 90% to 95% of the production of 5-HT in the body [116]. In ECs, 5-HT is formed out of the essential dietary amino acid tryptophan via the rate-limiting enzyme tryptophan hydroxylase 1 (TPH1) [62,117]. Further catalyzation with aromatic amino acid decarboxylase (AADC) leads to the production of 5-HT [118]. Serotonin from ECs is released predominantly at the baso-lateral side of the GI tract, where it exerts a multitude of effects by affecting almost every intestinal cell type expressed. Serotonin influences the frequency of GI contractions, modulates the intestinal immune response and once drained into the blood, regulates platelet aggregation [116,118,119]. Interestingly, a relationship has emerged between the colonic microbiome and 5-HT signaling. Indigenous spore-forming bacteria (Sp) present in the colon of mouse and man and known to be dominated by *clostridia* species have been shown to significantly promote colonic EC-serotonin biosynthesis in germ free (GF) mice [102], an effect consistent with other reports [101,120,121]. Interestingly, Sp microbiota-induced 5-HT production in ECs modulated GI motility and platelet function in these mice [102]. Several microbial-produced metabolites have been linked to this effect [102]. Of those, butyrate and propionate stimulated 5-HT release and/or enhanced TPH1 expression of ECs in vitro [101,102], while for the secondary bile acid deoxycholate, an effect on 5-HT was demonstrated in the colon, after intrarectal injection of deoxycholate in GF mice [102]. Deoxycholate has been shown to modulate colonic contractility thereby acting via TGR5 (GPBAR1), which is expressed on ECs [122].

Studies on ECs revealed expression profiles for GPCRs that sense bioactive fatty acids, of which, in the colon, most are microbial derived. Surprisingly, the highest enriched SCFA receptor in mice colonic ECs, purified by 5-HT antibody binding, was found to be Olfr78, followed by Olfr558, while FFAR2 (GPR43) and GPR35 were highly expressed but not enriched when compared to neighboring non-EC cells [95]. Interestingly, Olfr78 has also been reported to play a role in renin secretion and to act together with renal expressed FFAR3 (GPR41) in physiologically opposite roles to keep blood pressure balance [5,123]. Antibiotic treatment of Olfr78 KO mice suggested that SCFAs produced by intestinal microbiota and taken up in blood, were able to stimulate Olfr78 and FFAR3 in the afferent arteriole of the kidney [5,124]. Olfr78 responds to physiological levels of the SCFAs acetate and propionate, but not to butyrate [5]. As described in Section 3.2, OR51E2, the human orthologue of Olfr78, shows similar dose-response curves. Both receptors are found to be expressed along the GI tract, with a predominantly higher expression for Olfr78 in colon of mice, but this higher colonic expression was not observed in man [99]. In a model of DSS-induced colitis, Olfr78 KO mice showed higher levels of intestinal inflammation and altered expression of immune related- and inflammatory genes, including that of the cytokine IL-1β, known for its key role in colitis [99]. Although it is not yet known which EEC subtype is involved in the Olfr78-mediated immune modulatory responses (note that Olfr78 is also reported to be expressed by PYY producing L cells, see Section 4.2), a role for ECs seems conceivable, given 5-HT’s modulatory effects.

A direct effect of SCFAs for EC-mediated 5-HT release has been shown for Olfr558. Elegant experiments using a gut organoid model generated from ChgA-GFP mice revealed that Olfr558 acts as a branched chain fatty acid receptor for the irritant isovalerate, and likely senses information of pain and discomfort or/and the presence of pathogens via serotonergic signaling towards the brain [12]. Out of 30 relevant agonists, only the branched chain fatty acid isovalerate, and to a much lesser extent butyrate and isobutyrate, induced a response from individual ECs in organoids. As mentioned above, in young pigs, the orthologue of Olfr558, OR51E1, has been shown to almost fully co-localize with ChgA expression, indicating its presence on EECs. In addition, co-localization with 5-HT staining was observed in most cases [96].

In addition to FFAR2, and to GPR35, which senses kynurenic acid [107], Hcar2; (hydroxycarboxylic acid receptor 2; Niacr1; GPR109A), detecting butyrate and nicotinic acid [104], GPBAR1 (TGR5) the sensor for secondary bile acids and GPR132, which detects *N*-acylglycines and oxidized fatty acids [114] showed high expression levels in ECs from colon [95]. Additionally, Martin et al. found that in colonic ECs, FFAR4 showed similar high expression as FFAR2 and a moderate expression of GPR119 and GPR92 (LPA5), while FFAR3 was low expressed [103]. EC-studies comparing receptor expression levels from duodenal and jejunal ECs with those from colon uncovered that expression patterns of nutrient sensing receptors, including those of fatty acids, are location dependent. Lund et al. [95] state that ECs from the proximal GI tract do not express any FFARs but are indirectly activated by SCFAs via L cell released GLP-1. However, others have shown that ECs in the proximal GI tract in mouse express FFAR1, FFAR2, FFAR3 [58], FFAR4, GPR119, and GPR92 [103]. Our group has previously shown that nutrient-induced GLP-1 release by intestinal tissue segments is enhanced by 5-HT via a mechanism involving a 5-HT receptor expressed on EECs [125]. This also implies a cross-talk between EECs and ECs.

### 5.2. The 5-HT-Immune Axis

5-HT as released by ECs has a direct interaction with the intestinal immune system, of which several cell types are abundantly located in the direct vicinity of ECs. Serotonin-mediated functions seem to be tightly controlled, likely facilitated by the different 5-HT receptors, which are differentially expressed on 5-HT-responsive cells, and by the enzyme serotonin reuptake inhibitor (SERT), which inactivates 5-HT. Out of the 5-HT receptor (5-HTR) families, members of the 5-HTR 1–4 and 7 families are expressed in the gut and belong to the GPCRs, with an exception for 5-HTR3, and they signal via activation of different intracellular secondary messenger cascades [118].

Macrophages, dendritic immune cells (DCs), and T regulatory cells reside within the *lamina propria*, located immediately beneath the epithelial layer and they play crucial roles in maintaining intestinal immune homeostasis. Immune cells in the proximity of ECs are in a quiescent, anti-inflammatory state. Self-maintaining macrophages persist throughout adulthood and contribute to gut immune homeostasis [126]. Short-lived resident macrophages are constantly replenished by migratory monocytes from blood. When intestinal homeostasis is disrupted during pathogenic infections and (or) inflammatory conditions, entering blood monocytes will differentiate locally into macrophages and DCs with proinflammatory phenotypes and trigger an inflammatory cascade [118]. In vivo experimental studies have underlined 5-HT as a key proinflammatory molecule in gut inflammation [118]. Several studies report that 5-HT acts specifically on different types of immune cells through its activated 5-HTRs. Serotonin can potently enhance the proinflammatory effect of lipopolysaccharide (LPS), the major component of the outer membrane of gram-negative bacteria, as was shown in LPS-activated human-derived monocytes and peritoneal mice macrophages. Proinflammatory interleukins such as interleukin-1β (IL-1β) and IL-6 levels were largely increased by the combination of LPS and 5-HT, while 5-HT by itself only induced a minor induction [119,127]. In monocytes, this effect was mediated via activation of 5-HTR3, 5-HTR4, and 5-HTR7 subtypes. Serotonin solely, in the absence of LPS, has been shown to augment phagocytic activity of peritoneal mice macrophages thereby acting via nuclear factor kappa-light-chain-enhancer of activated B cells (NF-κB) and 5-HTR1A [128].

Animal KO studies have largely contributed to the recognition of 5-HTs immune-modulatory role. In a TPH1 knock out (TPH1(−/−)) colitis mice model, that lack 5-HT produced by ECs, it was found that macrophage infiltration, IL-1β, IL-6, and TNFα production and colitis-associated colonic tissue damage were significantly reduced compared to the control (TPH1(+/+)) mice [119]. Using a similar model, it was shown that TPH1(−/−) DCs produced less proinflammatory IL-12 than control DCs. Interestingly, CD4(+) thymus-derived lymphocytes (CD4(+) T cells) primed by TPH1(−/−) DCs produce reduced levels of IL-17 and interferon-γ, an effect that could be counteracted by adding 5-HT [129]. T helper 17 (Th17) cells are a specific subset of CD4(+) T cells that in gut protect the host from invading microorganisms. They are characterized by the expression of the transcription factor retinoic acid receptor-related orphan receptor gamma (RORγt) and once activated they produce the inflammatory cytokine IL-17 [130]. Intriguingly, secretory products from CD4(+) T cells interact with ECs to enhance the production of 5-HT in the gut via T helper 2 (Th2)-based mechanisms [131]. The IL-13 receptor α1, which was found to be expressed on ECs, seems to play an important role in this effect [131,132,133]. Such a positive feedback loop between T cells and ECs might partly explain the plasticity of 5-HT mediated immune-modulatory effects. Also in macrophages, 5-HT seems to fulfill opposite immune-modulatory roles, acting on one hand as a proinflammatory accelerator during pathogen invasion, while on the other hand contributing to a hyporesponsive environment for commensal microbes [130,134]. In addition, the anti-inflammatory effect of butyrate on resident intestinal immune cells is thought to contribute to the symbiotic relationship between beneficial commensals and the host [77].

**Figure 1:**
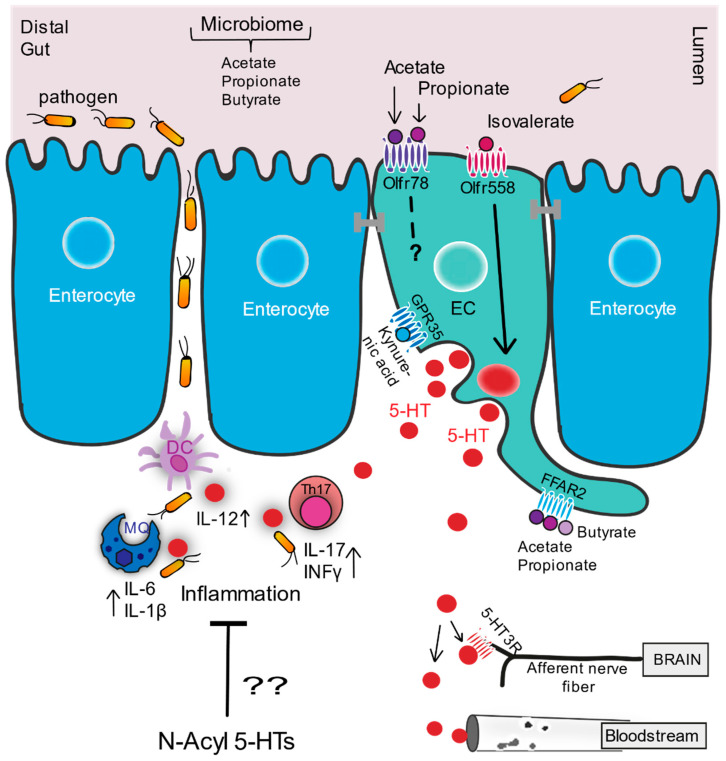
Serotonin signaling cascade in distal gut. Figure shows activation of serotonin by the branched chain fatty acid, isovalerate and microbial-produced short chain fatty acids (SCFAs), its interplay with the immune system, pathogens, and nervous system and its trafficking via the blood stream. Enterochromaffin cells (ECs; in green) situated within the epithelial lining (enterocytes in blue) of the gastrointestinal (GI) tract. Figure illustrates EC surface expression of olfactory receptors (Olfrs) and other GPCRs, showing their presumed orientation on the luminal versus basolateral membrane of ECs and their activation by specific fatty acids. The proinflammatory interaction of 5-HT with different immune cells is depicted during a proinflammatory response triggered by microbes that passage the epithelial lining during GI damage. The potential interaction of anti-inflammatory gut produced *N*-acyl serotonins (*N*-acyl 5-HTs) with the intestinal immune system is proposed. MQ: macrophage, DC: dendritic cell, Th17: T helper 17 cell, 5-HT: serotonin, 5-HTR: serotonin receptor.

Concluding, intestinal 5-HT is most well-known for its proinflammatory role in diverse pathologies of the gut, where it seems to further accelerate inflammatory conditions induced by varying immune triggers. Reports originating from animal models on inflammatory bowel disease (IBD), SERT KO, TPH1 KO models, and pathogen invasion studies as well as clinical data from Crohn’s disease patients have collectively contributed to this view [130,131,133,135,136,137]. Figure 1 shows the 5-HT signaling pathway, the FFA ligands, and corresponding receptors that trigger its release and subsequent interactions.

### 5.3. Fatty Acid-5-HT Conjugates with Immune-Modulatory and Anti-Oxidant Effects

While 5-HT predominantly exerts proinflammatory effects, emerging evidence points to the anti-inflammatory and anti-oxidant effects of its endogenous fatty acid-conjugates, *N*-acyl 5-HTs, present in the gut [38,39,40,41,138]. Our laboratory uncovered that docosahexaenoyl-serotonin (DHA-5-HT), the conjugate of 5-HT with the *n*-3 poly unsaturated long chain fatty acid (LC PUFA) DHA, has anti-inflammatory effects both in mouse macrophages as well as in human peripheral blood mononuclear cells (PBMCs), by attenuating release of key inflammatory mediators [40,41]. More specifically, in LPS-stimulated RAW264.7 mice macrophages, DHA-5-HT reduced, at concentrations of 100 nM to 500 nM, levels of prostaglandin PGE2 and of the cytokines IL-1β, IL-23 and IL-6 as well as expression of their corresponding genes [40]. By acting in an orchestrated way, these inflammatory mediators activate the IL-23-IL-17 signaling axis, the latter drives the development of pathogenic Th17 cells, a cell type involved in gut pathogenesis. Additionally, DHA-5-HT attenuated chemokine expression and migration of RAW264.7 cells [40]. In line with this, we found that in concanavalin A (ConA)-stimulated (human) PBMCs of healthy subjects, IL-17 cytokine, a typical Th17 pro-inflammatory mediator and C-C motif chemokine ligand 20 (CCL-20) release were inhibited by DHA-5-HT [41].

A series of six long chain fatty acid conjugates with 5-HT, namely palmitoyl-serotonin (PA-5-HT), stearoyl-serotonin (SA-5-HT), oleoyl-serotonin (OA-5-HT), arachidonoyl-serotonin (AA-5-HT), eicosapentaenoyl-serotonin (EPA-5-HT), and DHA-5-HT has been identified in intestinal tissue of mice, pigs and human colon [41,139]. Interestingly, in mice it was shown that levels of *N*-acyl serotonins formed in vivo could be modulated by the specific fatty acid composition of the diet. A high fish oil diet resulted in increased levels of DHA-5-HT and EPA-5-HT in gut, while the levels of other *N*-acyl serotonins from this series, like SA-5-HT, OA-5-HT, and AA-5-HT were decreased [139]. In vitro it was shown that the concentration of 5-HT did influence formation of *N*-acyl 5-HTs as well [139]. Following its initial discovery by Bisogno et al., AA-5-HT has been shown to act as a fatty acid amide hydrolase (FAAH) and a transient receptor potential cation channel subfamily V member 1 (TRPV1; also called vanilloid receptor 1) inhibitor [140,141]. However, while AA-5-HT, OA-5-HT, EPA-5-HT, and PA-5-HT possess FAAH inhibitory activity, though with similar IC50 values as their parental fatty acids, the *n*-3 DHA-5-HT- and 5-HT-saturated conjugate with stearic acid, SA-5-HT, lack this property. DHA-5-HT, in turn displayed the highest potency in inhibiting IL-17, in ConA-stimulated human PBMCs, while 5-HT and DHA showed no effects at this concentration [41]. Interestingly, *N*-acyl 5-HTs of long chain fatty acids were also identified in the human colon [41]. Altogether, this might indicate that the different *N*-acyl 5-HTs might act via different signaling pathways and/or receptors.

**Figure 2:**
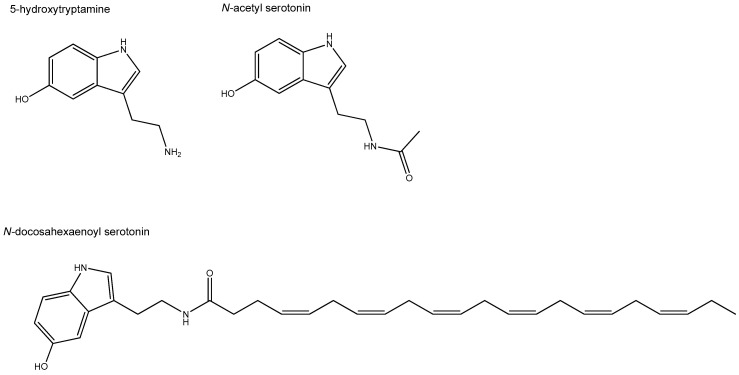
Chemical structures of serotonin (5-HT; 5-hydroxytryptamine) and its anti-inflammatory conjugates *N*-acetyl serotonin (NAS) and docosahexaenoyl serotonin (DHA-5-HT).

Furthermore, *N*-acyl-5-HTs with varying fatty acid moieties were reported to exert anti-inflammatory, anti-oxidant, and neuroprotective effects in a number of in vivo animal disease models [38,39,142,143,144,145]. In an inflammatory in vivo gut model, the SCFA conjugate of 5-HT, *N*-acetyl-5-HT (also called NAS), has been shown to prevent gut mucosal damage and inhibit programmed cell death following intestinal ischemia-reperfusion (IR) in rats [38]. Intestinal IR is a multifactorial pathophysiological process involving nonspecific damage of the gut, which can occur during trauma, sepsis, or shock. In this rat model, *N*-acetyl-5-HT downregulated IR-induced Toll-like receptor 4 (TLR-4), myeloid differentiation factor 88 (Myd88), tumor necrosis factor α (TNF-α), and receptor-associated factor 6 (TRAF6) expression in jejunum and ileum tissue [39]. *N*-acetyl-5-HT (NAS) is an endogenous precursor of melatonin and formed out of 5-HT. It is present in nanomolar concentration in human blood and seems capable to cross the blood-brain barrier [142]. NAS is approved by the U.S. Food and Drug Administration (FDA) for treatment of neurological disorders and stroke [142]. For the gut, its anti-oxidant properties have recently been assessed in a porcine epithelial IPEC cell line. Here, it was shown that activation of nuclear factor erythroid-2-related factor 2 (Nrf2) signaling was critical for the protective effect of NAS (at 100 µM) against oxidative stress [146]. Nrf2 signaling is key in cell survival in response to oxidative damage. Besides, NAS improved levels of tight junction proteins that were diminished by induced-oxidative damage [146]. Likewise, DHA-5-HT, at far lower concentrations, triggered Nrf2 pathway expression in LPS-activated macrophages. Using whole genome wide expression analysis followed by gene set enrichment analysis, the Nrf2 pathway was uncovered as most prominent upregulated pathway following stimulation of 1 µM DHA-5-HT [40]. Figure 2 shows the chemical structures of 5-HT and those of its fatty acid-conjugates that are of potential relevance for the gut.

Concluding, a specific functional role for endogenous *N*-acyl 5-HTs in intestinal health and gut homeostasis is still speculative; however, given their formation and presence in gut and their potent immune-modulatory and anti-oxidant effects, it seems conceivable that *N*-acyl 5-HTs might play physiological roles in resolving inflammation and oxidative stress (see Figure 1). It remains unclear to what extent the significance of conjugation mainly lies in the formation of these anti-inflammatory 5-HT derivatives or whether conjugation is to be considered a functional pathway aimed at modulating the pro-inflammatory activity of 5-HT itself. This warrants further investigation.

## 6. Future Perspectives

### 6.1. Other Bioactive Fatty Acid Conjugates with Potential Relevance for Entero-Endocrine Signaling

**Figure 3:**
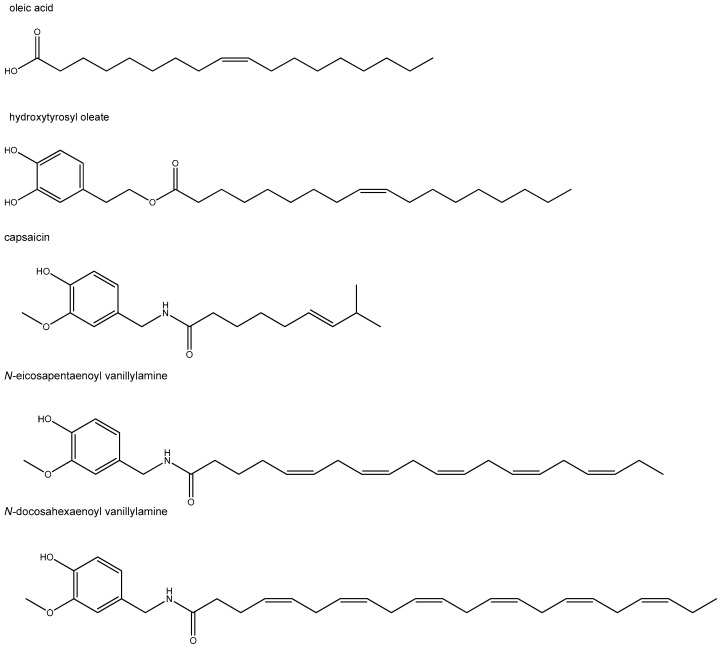
Chemical structures of oleic acid and its conjugate with hydroxytyrosyl, hydroxytyrosol oleate, and of capsaicin and its conjugated forms with EPA and DHA, *N*-eicosapentaenoyl vanillylamine (EPVA) and *N*-docosahexaenoyl vanillylamine (DHVA), respectively.

The discovery of fatty acid conjugates with specific bioactivities derived from endogenous amines and alcohols raised the question whether these structures could also be formed with molecules of exogenous, including dietary, origin. This search resulted in a number of potentially interesting compounds, with at least some of them showing interaction with the GLP-1-pancreatic signaling axis or (and) the immune system. For example, the conjugate of oleic acid with the flavonoid quercetin displayed the ability to induce insulin secretion via FFAR1 (GPR40) in a pancreatic cell line, and the combination of oleic acid with quercetin improved diabetic foot ulcers in patients [147,148]. A functional role for the FFAR1 receptor in pancreatic beta-cells and immune cells has earlier been evidenced. Another interesting oleic acid conjugate, hydroxytyrosyl oleate (see Figure 3 for its chemical structure), was found to possess immune-modulatory and antioxidant effects in vitro [149,150]. It was identified as a component of olive oil and its by-products. Noticeably, in contrast to the low oral bioavailability of the well-known bioactive olive oil constituent hydroxytyrosol, its more lipophilic oleic conjugate with tyrosol displayed good oral bioavailability. This raises the question whether enhanced flavonoid bioavailability might be a feature property of this class of fatty acid conjugates.

In several diabetic animal models, capsaicin, the major pungent component of red peppers, has been shown to improve glucose tolerance and increase insulin secretion. It is also reported that capsaicin exhibits anti-inflammatory effects [151]. Interestingly, the EPA and DHA *N*-acylamide conjugates of capsaicin, *N*-eicosapentaenoyl vanillylamine (EPVA) and *N*-docosahexaenoyl vanillylamine (DHVA), respectively, were found to be more potent than their parental molecules in attenuating inflammatory mediators and chemokines in a macrophage inflammatory cell line model [151]. This is in line with what was described in Section 5.3 for 5-HT-conjugates [40].

The EPA conjugate, EPVA, also elicited insulin secretion in pancreatic INS-1 832/13 β-cells, associated with raising intracellular Ca^2+^ and ATP concentrations [151]. An interesting observation was also that EPVA and DHVA had lost the pungent property of their parent molecule capsaicin. In Figure 3, the chemical structures of EPVA and DHVA are depicted.

Apparently, the ability to form fatty acid conjugates seems rather common in nature. As a consequence, it is conceivable that humans are exposed to these structures more than previously assumed through their diet or via synthesis in the GI tract by combining their molecular building blocks. However, more studies are warranted to gain insight into the relevance of these processes and their possible nutritional and (or) pharmacological applications in the context of GI EEC signaling and beyond.

### 6.2. Olfactory Receptors, Emerging Intestinal Fatty Acid Sensors with Potential for EEC Signaling

The significance of ORs for intestinal physiology has only quite recently emerged, with the number of receptors demonstrated to be involved being steadily on the rise [4,9,12,14]. Given their large variety, it is intriguing to further explore the roles of this large gene family in intestinal signaling.

The olfactory receptor family currently comprises of approximately 370- and 1000 functional ORs, in man and mouse, respectively. The majority of these still bears an orphan receptor classification, indicating that their endogenous ligand(s) has (have) not been determined. It seems conceivable that the plethora of fatty acid metabolites, either of microbial or dietary origin, will be, at least partly, sensed by the host. Their detection is crucial, not only as energy source but also to recognize and respond to potentially harmful molecules resulting from dysfunctional fermentation and (or) invading pathogens or, vice versa, to interact with commensal beneficial microbes. Olfr558, for example, was the first identified chemosensor able to detect the branched chain fatty acids, isovalerate and isobutyrate in gut [12]. Branched chain fatty acids, including isovalerate and isobutyrate constitute a minor part of the total fatty acid composition where they are present as a result of amino acid degradation. Interestingly, more ORs are known, exhibiting response profiles for fatty acids produced by the microbiome, but these ORs have not yet been linked to intestinal physiology [152].

Another interesting aspect to address is the potential physiological relevance of ORs for immune signaling, although the number of reports pointing towards such phenomena is still rather limited. An abundant number of gene transcripts for class I odorant receptors, the most ancient class of ORs conserved throughout evolution, were found to be expressed on five different types of human blood leukocytes, including bone-marrow-derived lymphocytes (B cells) and T cells [7]. Functional relevance for ORs has been shown for CD4+ T cells, of which trafficking abilities were largely diminished by odorant stimulation [17]. In addition, in lung macrophages, odorants enhanced both OR expression, monocyte chemoattractant protein-1 (MCP-1) production and macrophage migration, but only in the presence of inflammatory triggers [18]. As studies on OR functioning in immune cells are still very scarce and not associated with the intestinal immune system yet, further research should reveal how and if OR-chemosensing is involved in immune-modulatory processes in the gut and intermingled in the complex interaction of 5-HT and butyrate with the intestinal innate and adaptive immune system.

Emerging reports have provided first evidence for the influence of environmental factors on OR expression in the gut. In pigs, Priori et al. [96] showed that age, pathogen challenge, as well as dietary manipulations modulated *OR51E1* gene expression in GI tissues, an effect particularly related to the factors that affect complexity of the microbiota. Other studies also suggest an effect of dietary-associated obesity on OR expression. A comparison between obesity-prone rats and obesity-resistant rats that were both subjected for two weeks to either a HF or a LF diet, found differences in OR gene expression in duodenal enterocytes [153]. Furthermore, one of the studies originating from our own group showed that weight loss, induced by one year gastroplication in a group of morbidly obese people and accompanied by substantial weight loss, had beneficial effects on inflammatory and metabolic biomarkers. Interestingly, genome wide transcriptome analysis upon one year gastroplication, revealed that the orphan olfactory receptor OR2L8 appeared to be among the top highest significantly downregulated genes in duodenum [154].

### 6.3. Future Applications

In recent years, the importance of nutrition and the intestinal microbiome in GI physiology and energy metabolism has become increasingly apparent. Taste receptors for bitter compounds and FFAs in the gut have been shown to play prominent roles in signaling of nutrients, tastants, and microbial metabolites via essential insulin modulating gut hormones like GLP-1 [1,8,65]. Hence, the impending implications of modulating nutrient sensing seems to be large, given their effects on GLP-1, and underscores the huge potential of understanding the full spectrum of chemosensory signaling, including those of odorant receptors, in the gut. Understanding their role can lead to optimization of dietary guidelines for obese and T2D patients, healthier food products, or to novel therapeutic targets. Of particular interest seems the potential relevance of Olfr15 and Olfr544 in the pancreas [20,21,22]. These ORs are chemosensors for medium chain fatty acids, a group of nutritionally relevant FFAs, whose potential effects as signaling molecules [19,155] have received little attention so far. Further unravelling their specific roles and ligand specificity could have implications to control insulin homeostasis. Additionally, data for Olfr78 expression (mouse orthologue of OR51E2) on L cells and OR51E1-mediated GLP-1 release by nonanoic acid indicate a role for incretin signaling via these receptors [96,97].

Interestingly, in addition to obesity/T2D, many specific GI disorders seem to be related to intestinal enteroendocrine (dis)functioning. An increasing number of studies indicate that inflammatory bowel syndrome as well as irritable bowel disease might be (partly) linked to altered serotonergic signaling of ECs. The emerging evidence that Olfr78 and Olfr558, expressed by ECs, play a role in inflammation and pain sensation, respectively, could give rise to potentially novel nutritional, as well as pharmacological applications [12,99]. Fiber intake, gut microbial diversity and composition, as well as new sustainable protein sources (as a consequence of future dietary protein transition) might all affect EC-mediated 5-HT signaling via ORs by modulation of levels of SCFAs and BCFAs. Furthermore, more detailed knowledge about the function of 5-HT in the aetiology of inflammatory disorders of the gut and the specific potential role of its fatty acid conjugates in resolving inflammation could lead to novel drug development.

Another aspect that deserves particular attention is the role of OR51E2 and OR51E1 in cancer. While OR51E2 is intensively being studied for prostate cancer [16], the finding that OR51E1 is a marker for malignant enteroendocrine cells in intestinal cancer [10,11] is less well known. Overexpressed OR51E1 by malignant cells could serve as a specific biomarker or targeting OR51E1 might lead to novel therapies [10,11]. Intriguingly, gaining insight into their endogenous role and ligand specificity might give rise to dietary means to prevent early malignant behaviour of OR51E1 and OR51E2 expressing tissues or cells. Still puzzling is the broader ligand profile and corresponding sensitivity of these broadly expressed ORs, which raises the question as to whether different tissue or cellular functionalities might exist by virtue of ligand specific signaling in these cells or tissues. On the contrary, these ORs might mediate general physiological signals. Unravelling this issue could boost novel therapeutic as well as nutritional constrains.

Altogether, the studies outlined in this review underscore the significance of OR EEC signaling for GI physiology, however, at the same time they show how much still remains to be discovered about their functionality in relation to gut health. It seems we are only facing the beginning of the elucidation of the full spectrum of ORs, their ligand profiles as well as their potential diverse functional roles. Understanding their physiology in the interplay of the dietary-fatty acid-microbiome-EEC signaling axis might provide new leads urgently needed to combat the obesity pandemic or impact gastrointestinal disorders.

Concluding, this review highlights the versality of fatty acids as messengers in the GI tract and particularly focusses on interesting emerging players, including fatty acid sensors, with potential importance for 5-HT and GLP-1 signaling. Evolving research has revealed the relevance of intestinal hOR51E2/mOlfr78, hOR51E1/mOlfr558 and pancreatic mOlfr15 and mOlfr544 for energy- and insulin/glucagon metabolism, respectively, and their interaction with nutrients and/or microbial derived metabolites as MCFAs and SCFAs. In relation to gut health, the BCFA isovalerate, sensed by Olfr558, signals through the gut-brain axis via serotonergic signaling by enterochromaffin cells. Interestingly, while Olfr78 plays a prominent role in immune processes of an inflammatory gut disorder, environmental factors such as diet, obesity, age, pathogen challenge, and microbiota composition seem to alter OR expression of till this far unrecognized ORs. Furthermore, of particular interest are the fatty acid conjugates with serotonin and those of LCFAs such as oleic acid and DHA. These LCFA-conjugates exhibit anti-inflammatory, anti-oxidant, or insulin stimulatory properties. Overall, the interplay of bioactive fatty acids and their conjugates at diverse locations within the signaling cascade with EECs or immune cells points to their diverse functional relevance for intestinal physiology and hence for their impact for health and disease.

## Figures and Tables

**Table 1:** Fatty acid ligands with their GPCR receptors, tissue and cell (EC and/or L cell) expression, and potential function in 5-HT and GLP-1/PYY signaling in gut.

Ligand	GPCR	Expression in Gut	Function in Gut	Ref.
			Physiological Effects of Intervention	
Acetate, propionate lactate	mOlfr78	PYY & GLP-1 expressing cells; Colonic ECs; Entire GI tract	Microbiome	[78] ^L^; [5] ^L,F^; [79] ^L^; [94] ^L,E^; [95] ^E^; [99] ^E,F^
			Immune	
Acetate, propionate; palmitic acid, *N*-acetyl glutamic acid	hOR51E2	Entire GI tract	Unk.	[78] ^L^; [4] ^E^; [5] ^L^; [16] ^L^; [99] ^E^
Isovalerate > butyrate, isobutyrate	mOlfr558	Colonic ECs	5-HT signaling discomfort, pain	[12] ^L,E,F^; [95] ^E^
3- and 4-methyl-valeric acid > valeric acid, isovaleric acid, 2-methyl-valeric acid, nonanoic acid; Cyclobutanecarboxylic acid > 2-methylbutanoic acid	hOR51E1	ChgA, PYY & 5-HT expressing cells ^P^; GI tract, high in duo and stom ^P^; /Cecal EECs	Diet, Infection/GLP-1	[80] ^L^; [81] ^L^; [96] ^L,E,F^; [100] ^L^; [97] ^F^
Acetate, propionate > Butyrate	FFAR2	EC/EEC L	5-HT ^+^, Microbiota ^+^/ GLP-1, PYY	[101] ^F^; [102] ^F^ [89] ^F^; [103] ^E^; [58] ^E^; [66] ^E,F^; [68] ^L,E,F^
Valerate, caproate > acetate, propionate, butyrate	FFAR3	EC/EEC L	Unk./ GLP-1, PYY	[89] ^F^; [58] ^E^; [66] ^E,F^; [68] ^L,E,F^
Butyrate	GPR109A/ HCAR2	EC	Unk. ^#^	[104] ^L^; [105] ^#^; [106] ^F^; [95] ^E^
Kynurenic Acid	GPR35	EC	Unk.	[107] ^L^; [108] ^E^; [109] ^L^
MCFA/LCFA DHA/EPA ^s^, oleic acid ^s^ lauric acid ^s^, myristic acid ^s^, palmitic acid ^s^	FFAR1	EC/EEC L	Unk./GLP-1	[90] ^F^; [58] ^E^; [66] ^E,F^; [68] ^L,E,F^
unsaturated LCFAs, (α)(γ)( )-linolenic acid ^s^, palmitoleic acid ^s^	FFAR4	EC/EEC L	Unk./GLP-1	[103] ^E^; [66] ^E,F^; [91]; [68] ^L,E,F^
OEA ^s^, PEA ^s^, LEA ^s^, 16:0-LPC ^s^, 18:0-LPC ^s^, 18:1-LPC ^s^, (S)/(R)-*N*-oleoyltyrosinol ^s^, 1-OG ^s^, 2-OG ^s^, 5-HEPE ^s^	GPR119	EC/EEC L	Unk./GLP-1	[98] ^L^; [110] ^L^; [111] ^F^; [88] ^F^; [112] ^L,F^; [87] ^L,F^
*N*-acyl glycines (*N*-Palmitoylglycine ^s^)/oxidized fatty acids (9-HOPE ^s^)	G2A/GPR132	EC	Immune modulation	[113] ^L^; [95] ^E^; [114] ^L^; [115] ^F^

OR51E2 is a well-known marker for prostate cancer and responds to testosterone metabolites, e.g., 19-hydroxyandrostenedione; ^#^ GPR109A is expressed in epithelial cells in the colon and ileum and involved in intestinal inflammation, but no function has been described in relation to EEC signaling yet; ^+^ A specific role for FFAR2 has not been shown for SCFA-induced effects in ECs on 5-HT or TPH1. Abbrev.: DHA: docosahexaenoic acid; EPA: eicosapentaenoic acid; OEA: oleoyl ethanolamine; PEA: palmitoyl ethanolamine; LEA: linoleyl ethanolamine; 16:0-LPC: 1-palmitoyl-lysophosphatidylcholine; 18:0-LPC: 1-stearoyl-lysophosphatidylcholine; 18:1-LPC: 1-oleoyl-lysophosphatidylcholine; 2-OG: 2-oleoyl glycerol; 1-OG: 1-oleoyl glycerol; 5-HEPE: 5-Hydroxy-eicosapentaenoic acid; 9-HOPE: 9-hydroxyoctadecadienoic acid; ^p^: in pigs; duo: duodenum; stom: stomach;. m: mouse; h: human; ^s^: fatty acid (-conjugate) ligands displaying lowest IC50 values; ^L^: ligand; ^E^: expression; ^F^: function; Unk: Unknown.

**Table 2:** Fatty acid ligands with their corresponding ORs, their expression, and potential function in the pancreas.

Ligand	GPCR	Expression in Gut	Function in Pancreas	Ref.
Octanoic acid	Olfr115	Panc. β cells/MIN6	Insulin signaling	[20,21] ^E,F^
Unknown	Olfr821	Panc. β cells/MIN6	Unknown	
Azelaic acid	Olfr544	Pancreatic α-cells αTC1-9 cells	Glucagon secretion	[22] ^E,F^

^E^: expression; ^F^: function

## Data Availability

Not applicable.
